# The Role of MicroRNA in Migraine: A Systemic Literature Review

**DOI:** 10.1007/s10571-023-01387-9

**Published:** 2023-07-11

**Authors:** Olga Grodzka, Stanisław Słyk, Izabela Domitrz

**Affiliations:** 1 Department of Neurology, Faculty of Medicine and Dentistry, Medical University of Warsaw, Ceglowska 80, 01-809 Warsaw, Poland; 2 Doctoral School, Medical University of Warsaw, Banacha 1A, 02-097 Warsaw, Poland

**Keywords:** Migraine, microRNA, Primary headache disorders, Neuroinflammation, Biomarker

## Abstract

Migraine is a common primary headache disorder, affecting about 14% of the population. Importantly, it was indicated as the second cause of disability globally and the leading cause among young women. Despite the widespread prevalence, migraine remains underdiagnosed and undertreated. The possible solution may be microRNAs—small, non-coding molecules. Until now, multiple studies have shown the great value of microRNA in both the diagnosis and treatment of different human diseases. Furthermore, a significant role in neurological disorders has been suggested. Little research regarding the utility of microRNA in migraine has been conducted, however, the results so far appear to be promising. We performed an electronic article search through PubMed and Embase Database to further explore the topic. After the analysis, according to PRISMA 2020 guidelines, we included 21 studies. The dysregulation was observed in migraine in general, as well as in different types and phases; thus, miRNAs emerge as promising diagnostic biomarkers. Additionally, some studies showed the influence of the intervention with miRNA levels on neuroinflammation and the expression of peptides, which are crucial in migraine pathogenesis. This review aims to summarize the current knowledge about the role of miRNAs in migraine and encourage to further research in this field.Kindly check and confirm the edit made in the title.I checked and confirm.

**Graphical Abstract:**

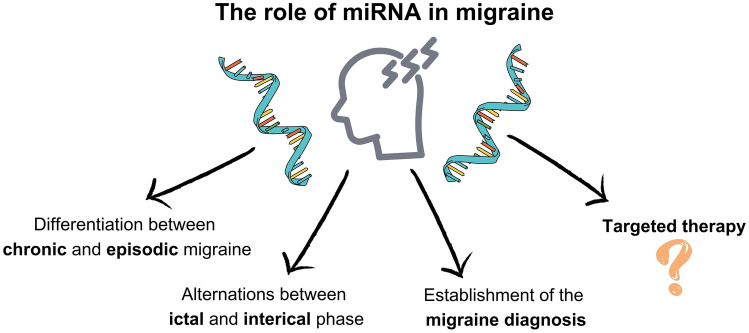

## Introduction

### Background Information About Migraine

According to the International Classification of Headache Disorders, 3rd edition (ICHD-3), migraine is a common primary headache disorder with two major types: migraine with aura and migraine without aura (Headache Classification Committee of the International Headache Society (IHS) The International Classification of Headache Disorders, 3rd edition 2018). The latter type occurs more commonly and is present in about 75% of migraineurs (Pescador Ruschel and De Jesus 2022). Importantly, the global prevalence of migraine was estimated at 14% (Stovner et al. 2022). Moreover, based on the Global Burden of Disease Study 2019, migraine was indicated as the second cause of disability overall and first in female patients under 50 years old (Global burden of 369 diseases and injuries in 204 countries and territories, 1990–2019: a systematic analysis for the Global Burden of Disease Study 2019 2020).

Although the prevalence of migraine is widespread, the disease remains constantly underdiagnosed and, thus, undertreated (Eigenbrodt et al. 2021). The final diagnosis is made on clinical criteria established by the ICHD-3 (Headache Classification Committee of the International Headache Society (IHS) The International Classification of Headache Disorders, 3rd edition 2018) and includes headache characteristics and associated symptoms (Silberstein 2004). Despite multiple studies to search for an ideal biomarker for migraine, a remarkable achievement in this field is still lacking (Ferreira et al. 2021).

### Biosynthesis and Function of microRNA

MicroRNAs (miRNAs) are small, non-coding ribonucleic acid (RNA) molecules that control messenger RNA (mRNA) expression (Hill and Tran 2021), either by inhibiting the translation or promoting the degradation (Correia de Sousa et al. 2019). The function of miRNAs, based on negative gene regulation, is to influence multiple genetic pathways, including cell proliferation, apoptosis, and metabolism in eucaryotic organisms (Mishra et al. 2016; Kolodziej et al. 2022).

However, starting from the beginning, the initial step of miRNA biogenesis is the synthesis of primary miRNA (pri-miRNA), which can be transcribed by RNA polymerase II or otherwise derived from introns of protein-coding genes (Krol et al. 2010). Secondly, the Drosha complex, located in the cell nucleus, transforms pri-miRNA into hairpin-structured RNA, called precursor miRNA (pre-miRNA) (Matsuyama and Suzuki 2019). Subsequently, the process moves to the cytoplasm, where the pre-miRNA molecule is processed into a miRNA duplex by another enzyme with a ribonuclease activity named Dicer (Ha and Kim 2014). An RNA-induced silencing complex (RISC) forms from loading a miRNA duplex into an Argonaute protein. One of the strands of the formed molecule, the passenger strand, is discarded, while the other, the guide strand, represents the mature miRNA (Lin and Gregory 2015). Usually, the 5ʹ end of miRNA is involved in the repression or degradation of complementary mRNA by binding to its 3’ end. However, there are other mechanisms of miRNA biosynthesis independent of Drosha and Dicer, resulting in the creation of a miRNA-mRNA complex containing the 3ʹ end of miRNA (Saliminejad et al. 2019; Helwak et al. 2013). We featured the information about the involved miRNA’s end in the analyzed studies whenever it was mentioned by the researchers in the original study.Please check and confirm that the authors and their respective affiliations have been correctly identified and amend if necessary.I checked and confirm.

Until now, over 2500 different miRNAs have been discovered in the human body (Çakmak and Demir 2020); therefore, they have been suggested as both diagnostic biomarkers and novel therapeutic targets (Ho et al. 2022). Continuous research to analyze the role of miRNAs in humans is being conducted. The potential role in the pathogenesis, diagnosis, and treatment of multiple diseases, such as oncological (Lee and Dutta 2009), cardiological (Grodzka et al. 2022; Procyk et al. 2022), and autoimmune diseases (Khodakarimi et al. 2021), has been extensively studied. Furthermore, miRNAs were suggested to be crucial in neurological disorders (Kamal et al. 2015).

### The Putative Role of microRNA in Migraine

**Fig. 1:**
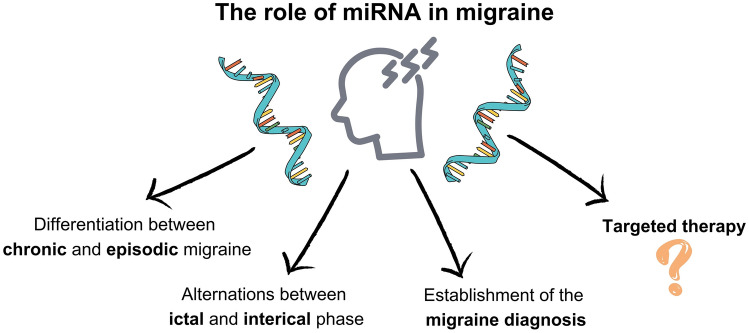
A graphical presentation summarizing the putative role of miRNAs in migraine management. miRNA, microRNA; RNA, ribonucleic acid

Despite many advances in exploring the pathogenesis of migraine, there is still a lack of specific diagnostic biomarkers (Gallelli et al. 2017). Thus, miRNAs may be a breakthrough in the diagnosis and, prospectively, the targeted therapy. The involvement of epigenetic mechanisms has been demonstrated in several studies regarding migraine pathophysiology (Ebahimzadeh et al. 2021; Fila et al. 2019). miRNAs have been shown to inhibit the function of proteins responsible for the development of migraine pain (Fila et al. 2022). Moreover, the dysregulation of several miRNAs has been observed in migraineurs compared to healthy individuals and in different types and phases of the disease (Tana et al. 2017). Therefore, microRNAs emerge as promising biomarkers in migraine diagnosis, and, by extension, the opportunity to discover novel targets for specific therapy has appeared (Gazerani 2019). This study aims to summarize the current knowledge about the role of miRNA in migraine (Fig. 1).

## Methods

**Fig. 2:**
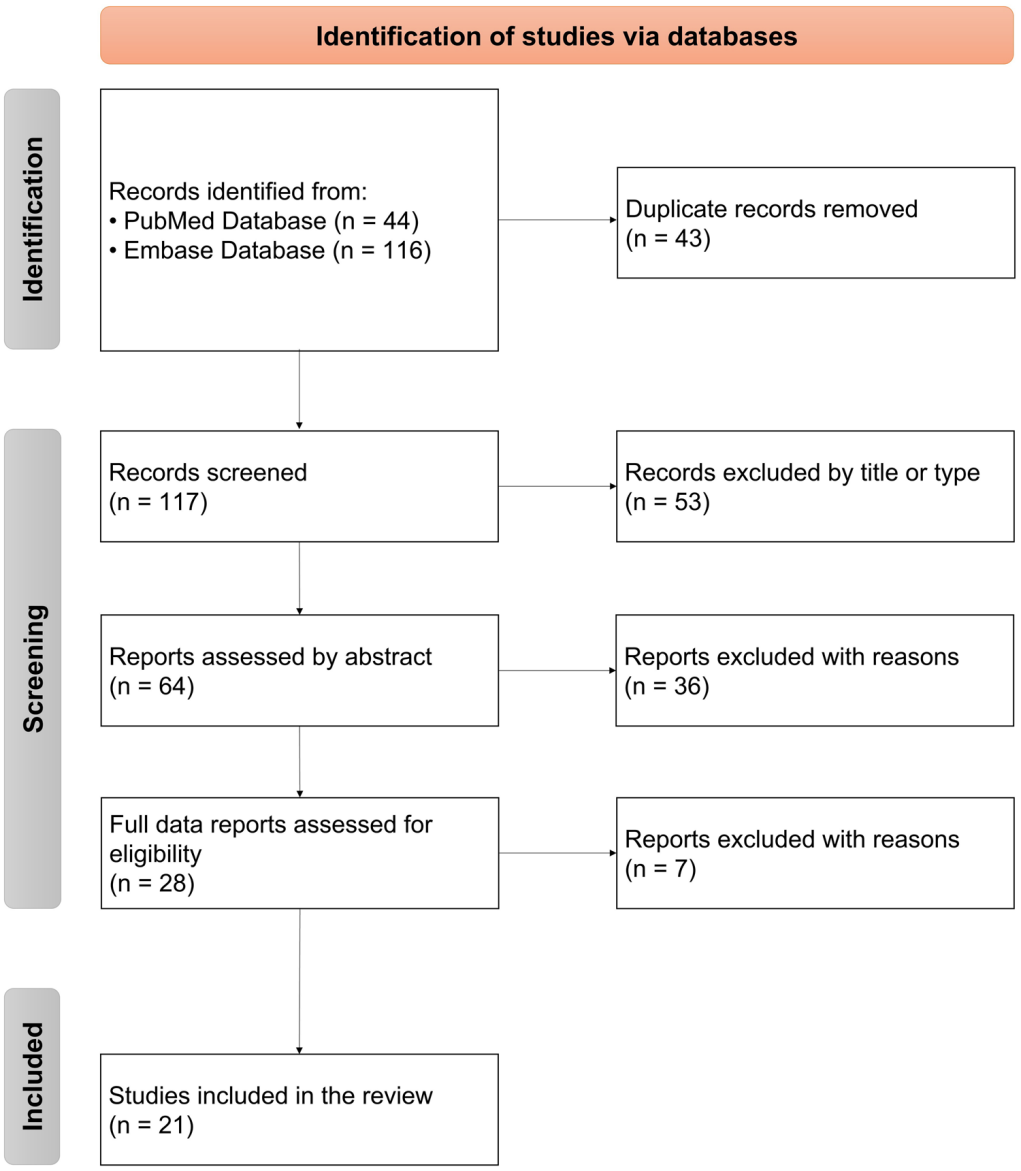
Flow diagram of the selection process according to the guidelines of Preferred Reporting Items for Systemic Reviews and Meta-analyses (PRISMA 2020); *n* number of studies

According to the guidelines of Preferred Reporting Items for Systemic Reviews and Meta-analyses (PRISMA 2020) (Page et al. 2021) the systemic review was performed (Fig. 2), along with an electronic article search through PubMed Database and Embase Database. The search strategy in both databases was as follows: (migraine) AND (microRNA OR miRNA).

### Inclusion and Exclusion Criteria

The inclusion and exclusion criteria were applied to include only the most adequate research. Apart from the articles, due to limited data, we decided to include conference abstracts, for which we followed generally agreed recommendations to choose studies only of the highest quality (Scherer and Saldanha 2019). The primary research, including data collected directly by the researchers, such as clinical or cohort studies and case reports, were allowed. The involved studies assessed the levels of microRNA in human or animal models, and the study group was defined as migraineurs or an animal model of migraine with or without any intervention.

The reviews, systemic reviews, meta-analyses, letters to the editors, and commentaries were not included. We excluded publications in which patients were affected by different diseases than migraine and preclinical trials without an established experimental model of migraine. Moreover, studies focusing on biomarkers other than miRNA were naturally rejected. We did not consider the studies written in languages other than English.

### Selection Process

The initial search with the abovementioned keywords resulted in considering 44 articles from PubMed Database and 116 from Embase Database. We removed the duplicate records, limiting the list to 117 different pieces of research. Subsequently, 53 studies were excluded because of the inadequate article type or by the title. After the assessment by the abstract, 36 studies did not meet the inclusion criteria and hence, did not present a direct contribution to the review topic. The full text of the remaining 28 studies was analyzed. However, 6 were publications duplicating the results regarding miRNA in migraine from another article, while one was excluded based on inclusion and exclusion criteria. Finally, the provided selection left us with 21 appropriate studies.

## MicroRNAs in Patients Suffering from Migraine

Until now, the knowledge about the role of miRNA in migraine remains insufficient. To analyze the problem more thoroughly, we divided the included research into the following parts: (i) miRNAs as diagnostic biomarkers in migraine patients, (ii) the role of miRNAs in chronic migraine; (iii) miRNAs in migraine with the specification of ictal or interictal phase, and (iv) the role of miRNAs in preclinical studies.

### miRNAs as Diagnostic Biomarkers in Migraine Patients

Gallardo et al*.* (2023) assessed the levels of different miRNAs in non-menopausal women in comparison to healthy controls. The upregulation of 59 and downregulation of 132 different miRNAs have been shown. The Random Forrest method for classification, including the features selecting algorithm, has been used to indicate the most significant biomarkers in differentiation between migraine patients and healthy controls. The three miRNAs: miR-342-5p, miR-532-3p, and miR-758-3p have been proven to present the highest value in migraine diagnosis. The dysregulation of multiple miRNAs was analyzed by another research group (Liu et al. 2022), which conducted a study on patients suffering from migraine without aura. The results showed 68 upregulated and 104 downregulated miRNAs as compared to healthy controls, which may be considered as potential biomarkers. Additionally, they analyzed acupuncture treatment's influence on migraine patients’ miRNA levels. After performing a true acupuncture treatment, the dysregulation of 12 different miRNAs appeared, not observed after sham acupuncture treatment.

Cheng et al*.* (2018) demonstrated that levels of miR-155, miR-126, and let-7 g were significantly higher in migraine patients as compared to healthy individuals. Moreover, patients suffering from migraine with aura presented higher levels of the abovementioned miRNAs than those without aura; however, the difference was not statistically significant. Furthermore, authors have shown a positive correlation between miRNA levels and syncope frequency among migraineurs. Tafuri et al*.* (2015) provided a comparison of different miRNA levels in migraineurs and healthy individuals. The upregulation of miR-27b was observed in the study group as compared to the control group, while levels of miR-181a, miR-22, and let-7b were significantly decreased in the plasma of patients suffering from migraine. However, in the peripheral blood monocytes, similar alterations were observed only concerning the expression of two latter miRNAs. Another analysis was held by Yasin et al*.* (2023), who compared the levels of specific miRNAs indicated as targets for the *CHRNA7* gene. *CHRNA7* encodes proteins involved in systemic inflammatory response with a potential role in migraine pathogenesis. Only the expression of miR-3158-5p was observed to be significantly downregulated in migraineurs as compared to healthy individuals; thus, it may be substantial for migraine diagnosis. However, the type of disease appeared to be irrelevant. Zhai et al. (2018) compared migraine patients to healthy controls and demonstrated a decrease of miR-30a in the study group. Furthermore, the *CALCA* (calcitonin-related polypeptide alpha) gene was identified as a target gene for the investigated miRNA. The elevated miR-30a level correlated negatively with CALCA expression. Therefore, the researchers concluded that the upregulation of miR-30a may relieve migraine by the degradation of CALCA. All studies discussed in this section with additional information are summarized in Table 1.

**Table 1:** Summary of recent studies regarding the role of miRNAs in migraine diagnosis

References	Year	Population	Comparison	miR	Outcome	Methodology
Gallardo et al. (2023)	2023	20 female MIG pts	12 female HC	miR-342-3p miR-532-3p miR-758-3p miRs as a whole	59 overexpressed miRs 132 underexpressed miRs ↓ miR-342-3p, miR-532-3p, miR-758-3p in MIG pts as compared to HC	miRs in PBMC by microarray
Liu et al. (2022)	2023	63 MIG pts without aura before and after ACP	32 HC	miRs as a whole	68 upregulated and 104 downregulated miRs in MIG pts as compared to HC 8 upregulated and 4 downregulated miRs in MIG pts after ACP as compared to sham ACP	miRs from serum exosomes by small RNA-Seq and qPCR
Cheng et al. (2018)	2018	30 MIG pts	30 HC	miR-155 miR-126 let-7 g	↑ miR-155, miR-126, let-7 g in MIG pts as compared to HC positive correlation between miR-155 and miR-126 levels and syncope frequency in MIG pts	miRs in plasma by qPCR
Tafuri et al. (2015)	2015	15 female MIG without aura pts	13 HC	miR-22 miR-27b miR-181a let-7b	↑ miR-27b in MIG pts as compared to HC ↓ miR-22, miR-181a, let7b in MIG pts’ plasma as compared to HC ↓ miR-22, let7b in MIG pts’ PBMC as compared to HC	miRs in plasma and PBMC by qPCR
Yasin et al. (2023)	2023	102 MIG pts (43 with aura, 59 without aura)	120 HC	miR-3158-5p	↓ miR-3158-5p in MIG pts as compared to HC no correlation between MIG pts with and without aura	miRs in blood by qPCR
Zhai and Zhu (2018)	2018	MIG pts	HC	miR-30a	↓ miR-30a in MIG pts as compared to HC correlation between ↓ CALCA and ↑ miR-30a	miRs in blood by qPCR CALCA in blood by WB

↑, increase; ↓, decrease

*ACP* acupuncture; *CALCA* Calcitonin Related Polypeptide Alpha; *HC* healthy controls; *MIG* migraine; *miR* microRNA; *miRs* microRNAs; *PBMC* peripheral blood mononuclear cells; *pts* patients; *qPCR* quantitative polymerase chain reaction; *RNA* ribonucleic acid; *Seq* sequencing; *WB* western blotting

### The Role of miRNAs in Chronic Migraine

Ahmad et al*.* (2022) investigated patients suffering from chronic migraine with medication overuse and compared them to migraineurs with an episodic type of the disease and healthy individuals. The former group showed increased levels of miR-34a-5p, miR-382-5p, and peptides: CGRP (calcitonin gene-related peptide) and PACAP (pituitary adenylate cyclase-activating peptide) as compared to the others. Noteworthily, 2 months after conducting the detoxification protocol in the study group, miR-34a-5p expression, as well as levels of both CGRP and PACAP, were decreased. Another interesting study was conducted by Gallardo et al*.* (2018), who analyzed the differential expression of multiple miRNAs in patients suffering from migraine in comparison to healthy individuals. The dysregulation of 41 miRNAs was observed in migraineurs in general. More specifically, 35 different miRNAs were altered in patients with chronic migraine, while patients suffering from episodic migraine presented the dysregulation of 24 miRNAs. Therefore, miRNAs may be useful in distinguishing between chronic and episodic migraine. A similar study, with a general assessment of deregulated miRNAs, was obtained by Burstein et al*.* (2014), who measured the levels of multiple miRNAs in migraineurs and compared them to healthy controls. The researchers observed the dysregulation of 27 different miRNAs out of 726 miRNAs assessed in patients with chronic migraine in comparison to the control group. In more detail, 16 miRNAs were upregulated, while 11 miRNAs appeared to be downregulated. A slightly different analysis was held by De Icco et al*.* (2020), who compared the patients suffering from chronic migraine before and after the injection of erenumab (anti-CGRP monoclonal antibody). As it appeared, the erenumab administration resulted in lower levels of miR-34a-5p and miR-382-5p in the migraineurs. However, the response rate to the treatment was shown to be irrelevant to the expression of analyzed biomarkers.

The opposite results were obtained by another research group (Vila-Pueyo et al. 2014). To investigate the patients suffering from migraine, they divided the study group into four cohorts: (i) chronic migraine patients with aura, (ii) chronic migraine patients without aura, (iii) episodic migraine patients with aura, and (iv) episodic migraine patients without aura. After determining the miRNA expression profiles, all possible comparisons were conducted within the cohorts and each cohort was compared to healthy controls. No statistically significant differences in any of the comparisons were observed. Noteworthily, the research was held on very small groups of patients. All studies discussed in this section with additional information are summarized in Table 2.

**Table 2:** Summary of recent studies regarding the role of miRNAs in chronic migraine

References	Year	Population	Comparison	miR	Outcome	Methodology
Ahmad et al. (2022)	2022	13 CM with medication overuse pts	21 EM pts 17 HC	miR-34a-5p miR-382-5p	↑ miR-34a-5p, miR-382-5p, CGRP, PACAP in CM pts as compared to EM pts and HC positive correlation between miR-34a-5p, CGRP, PACAP, and headaches frequency	miRs in PBMC CGRP, PACAP in serum
Gallardo et al. (2018)	2018	18 CM pts 22 EM pts	22 HC	miRs as a whole	41 dysregulated miRs in MIG pts as compared to HC (35 in CM pts, 24 in EM pts)	miRs in PBMC by microarray
Burstein et al. (2014)	2014	27 CM pts	17 HC	miRs as a whole	16 upregulated and 11 downregulated miRs in CM pts as compared to HC	miRs in periosteum
De Icco et al. (2020)	2020	40 CM pts after anti-CGRP-treatment	40 CM pts before anti-CGRP-treatment	miR-34a-5p miR-382-5p	↓ miR-34a-5p, miR-382-5p after anti-CGRP-treatment	miRs in PBMC by qPCR
Vila-Pueyo et al. (2014)	2014	10 CM pts (5 with aura, 5 without aura) 10 EM pts (5 with aura, 5 without aura)	5 HC	miRs as a whole	No differences in any comparison performed	miRs in PBMC

↑, increase; ↓, decrease

*CGRP* calcitonin gene-related peptide; *CM* chronic migraine; *EM* episodic migraine; *HC* healthy controls; *MIG* migraine; *miR* microRNA; *miRs* microRNAs; *PACAP* Pituitary adenylate cyclase-activating peptide; *PBMC* peripheral blood mononuclear cells; *pts* patients; *qPCR* quantitative polymerase chain reaction; *RNA* ribonucleic acid

### miRNAs in Migraine with a Specification of Ictal or Interictal Phase

Aczél et al*.* (2022) assessed the levels of multiple miRNAs in patients suffering from migraine and compared them to healthy controls. Importantly, migraineurs were divided into two groups: (i) patients in the ictal phase and (ii) patients in the interictal phase. In comparison to healthy individuals, the first group presented the upregulation of 22 miRNAs and downregulation of 9, while in the second group, 14 miRNAs were overexpressed and 17 were underexpressed. Furthermore, the differential expression of 25 miRNAs was observed while comparing the migraineurs from both groups. Similarly, Chen et al*.* (2021) to investigate the patients presenting reversible cerebral vasoconstriction syndrome, divided the study group into three cohorts: (i) migraineurs in the ictal phase, (ii) migraineurs in the interictal phase, and (iii) patients without a migraine history. Three miRNAs (let-7a-5p, let-7b-5p, let-7f-5p) were upregulated in the former group as compared to the two others. On the contrary, the downregulation of miR-130b-3p was observed in the same study cohort. Another interesting research was held by Andersen et al*.* (2016), who assessed levels of multiple miRNAs in migraine patients including the distinction between ictal and interictal phases. The study showed the dysregulation of 32 different miRNAs, and the authors selected four for further investigation. The levels of miR-34a-5p, miR-29c-5p, and miR-382-5p were increased in an ictal phase of the disease while compared to healthy controls. However, in the interictal phase, only expression of the latter miRNA remained significantly elevated in comparison to healthy individuals.

A slightly different study was conducted by Gallelli et al*.* (2019), who performed all measurements during the ictal phase of the disease. The research group analyzed the levels of miR-34a-5p and miR-375 in pediatric patients suffering from migraine without aura and compared them to healthy controls. The upregulation of both miRNAs has been observed in the study group in comparison to healthy individuals. Subsequently, the study group was divided into two equal subgroups: (i) receiving NSAIDs (non-steroid anti-inflammatory drugs) or acetaminophen and (ii) without any pain-relieving treatment. It was shown that treating with NSAIDs resulted in lower miRNA levels in comparison to non-treated individuals. Greco et al*.* (2020) conducted a research to compare the migraineurs of different types. However, on the contrary to the previous study, all measurements were made only in the interictal phase of the disease. The levels of miR-34a-5p and miR-382-5p, as well as CGRP release, appeared to be increased in patients suffering from chronic migraine with medication overuse compared to episodic migraine patients. Nevertheless, after the analysis, including correction for sex, age, and disease duration, only the abovementioned miRNAs expression was significantly higher, with no relevant deregulation in the CGRP level. Furthermore, performing the in-hospital detoxification protocol in patients with chronic migraine resulted in decreased levels of both miRNAs and CGRP at the 2-month follow-up. All studies discussed in this section with additional information are summarized in Table 3.

**Table 3:** The summary of recent studies regarding the role of miRNAs in migraine with the specification of ictal and interictal phase

References	Year	Population	Comparison	miR	Outcome	Methodology
Aczél et al. (2022)	2022	16 MIG pts (IP) 8 MIG pts (IIP)	12 HC	miRs as a whole	22 upregulated and 9 downregulated miRs in MIG (IP) as compared to HC 14 upregulated and 17 downregulated miRs in MIG (IIP) as compared to HC 15 upregulated and 10 downregulated miRs while comparing the IP to IIP	miRs in PBMC by small RNA-Seq
Chen et al. (2021)	2021	30 MIG pts (IP)	30 HC 30 MIG pts (IIP)	let-7a-5p let-7b-5p let-7f-5p miR-130b-3p	↑ let-7a-5p, let-7b-5p, let-7f-5p, ↓ miR-130b-3p in MIG (IP) as compared to MIG (IIP) and HC	miRs in plasma by qPCR
Andersen et al. (2016)	2015	16 MIG pts in cohort 1 (8 in IP, 8 in IIP) 12 MIG pts in cohort 2	8 HC in cohort 1, 8 HC in cohort 2	miR-34a-5p miR-29c-5p miR-382-5p miR-26b-3p miRs as a whole	32 dysregulated miRNAs in MIG pts as compared to HC ↑ miR-34a-5p, miR-29c-5p, miR-382-5p in MIG pts (IP) as compared to HC ↑ miR-382-5p in MIG pts (IIP) as compared to HC	miRs in serum by qPCR
Gallelli et al. (2019)	2019	24 MIG (IP) without aura pediatric pts	12 HC	miR-34a-5p miR-375	↑ miR-34a-5p, miR-375 in MIG pts as compared to HC	miRs in serum and saliva by qPCR
Greco et al. (2020)	2020	28 CM (IIP) pts with medication overuse	27 EM pts	miR-34a-5p miR-382-5p	↑ miR-34a-5p, miR-382-5p, CGRP in CM pts as compared to EM pts ↓ miR-34a-5p, miR-382-5p in CM pts as compared to EM pts (after detoxication)	miRs in PBMC by qPCR CGRP in plasma by ELISA

↑, increase; ↓, decrease

*CGRP* calcitonin gene-related peptide; *CM* chronic migraine; *ELISA* enzyme-linked immunosorbent assay; *EM* episodic migraine; *HC* healthy controls; *IIP* interictal phase; *IP* ictal phase; *MIG* migraine; *miR* microRNA; *miRs* microRNAs; *PBMC* peripheral blood mononuclear cells; *pts* patients; *qPCR* quantitative polymerase chain reaction; *RNA* ribonucleic acid; *Seq* sequencing

### The Role of miRNAs in Preclinical Trials

Greco et al*.* (2022) designed an experimental model of chronic migraine by treating laboratory rats with nitroglycerin (NTG). The levels of miR-155-5p, miR-34a-5p, and miR-382-5p were significantly increased in rats with induced chronic migraine as compared to negative controls. Moreover, after administering the olcegepant (CGRP-receptor-antagonist), the abovementioned effects were reversed. The two latter miRNAs had been proven to be positively correlated with CGRP, a peptide with crucial meaning for migraine pathogenesis. Similarly, another research group (Wen et al. 2021) established an animal model of chronic migraine by NTG administration. The level of miR-155-5p appeared to be increased in mice treated with NTG as compared to healthy controls. The injection of miRNA-antagonist alleviated neuroinflammation, microglia activation, and CGRP-level, whereas mice treated with miRNA-agonist presented opposite effects. Furthermore, the research group analyzed the role of silent information regulator 1 (SIRT1), indicated as a target for miR-155-5p, which had been shown to alleviate neuropathic pain. According to the expectations, the activation of SIRT1 caused similar effects as the inhibition of miR-155-5p, while the inhibition of SIRT1 acts likewise as the agonist of miR-155-5p. Consistently, the elevated level of miR-155 in the migraine model was demonstrated by Greco et al*.* (2019), who designed the experimental animal model by administration of NTG. Along with miRNA expression, CGRP, and substance P levels appeared to be increased as compared to negative controls. Furthermore, the injection of URB937, a peripheral fatty-acid amide hydrolase, reversed the observed effects.

Interesting results were obtained by Zhang et al*.* (2020), who established the experimental model of migraine by electrical stimulation of the trigeminal ganglion in rats. Compared to the healthy controls, the rats in the study group presented higher levels of miR-34a-5p. However, intervention with electrical acupuncture resulted in the downregulation of the miRNA. Furthermore, the level of miR-34a-5p positively correlated with the levels of inflammatory cytokines and negatively with SIRT1 expression. Finally, Zhang et al*.* (2021) investigated the role of miR-34a-5p in the inflammatory response in rats. The upregulation of miR-34a-5p resulted in higher levels of inflammatory cytokines and increased release of CGRP. The opposite effect was reached by the inhibition of the studied miRNA. Therefore, miR-34a-5p was suggested to have a potential role in the pathogenesis of inflammation and pain during the migraine onset. All studies discussed in this section with additional information are summarized in Table 4.

**Table 4:** The summary of the recent preclinical studies regarding the role of miRNAs in migraine

References	Year	Population	Comparison	miR	Outcome	Methodology
Greco et al. (2022)	2022	6 CM model anti-CGRP-treated rats	6 CM model rats 6 NC rats	miR-155-5p miR-34a-5p miR-382-5p	↑ miR-155-5p, miR-34a-5p, miR-382-5p in CM model rats as compared to NC-rats ↓ miR-155-5p, miR-34a-5p, miR-382-5p in CM model rats after anti-CGRP-injection	miRs in medulla-pons, CSC, and TGs by qPCR
Wen et al. (2021)	2022	CM model mice CM model anti-miR-155-5p-treated mice CM model SRT1720-treated mice	NC mice CM model ago-miR-155-5p-treated mice CM model EX527-treated mice	miR-155-5p	↑ miR-155-5p in CM model mice as compared to NC mice ↓ inflammation and CGRP in CM model mice after anti-miR or SRT1720 administration ↑ inflammation and CGRP in CM model mice after ago-miR or EX527 administration	miRs in TGs by qPCR ICs and CGRP in TGs by ELISA, IF, and WB
Greco et al. (2019)	2019	CM model rats URB937-treated CM model rats	NC rats NC-treated CM model rats	miR-155	↑ miR-155, CGRP, SP in CM model mice as compared to NC mice ↓ miR-155, CGRP, SP in CM model mice after URB937 administration	miR, CGRP, SP in TGs
Zhang et al. (2020)	2020	10 MIG model rats	10 NC rats 10 MIG model rats after ACP	miR-34a-5p	↑ miR-34a-5p in MIG model rats as compared to NC-rats ↓ miR-34a-5p in MIG model rats after ACP	miRs in TGs by qPCR and WB
Zhang et al. (2021)	2020	miR-34a-5p-overexpressing rats	miR-34a-5p-underexpressing rats NC-treated rats	miR-34a-5p	↑ ICs and CGRP release after miR-34a-5p upregulation ↓ ICs and CGRP release after miR-34a-5p downregulation	ICs and CGRP in TGs by ELISA

↑, increase; ↓, decrease

*ACP* acupuncture; *ago-miR* miRNA’s agonist; *anti-miR* miRNA’s antagonist; *CGRP* calcitonin gene-related peptide; *CSC* cervical spinal cord; *CM* chronic migraine; *ELISA* enzyme-linked immunosorbent assay; EX527, SIRT1-inhibitor; *HC* healthy controls; *ICs* inflammatory cytokines; *IF* immunofluorescence analysis; *MIG* migraine; *miR* microRNA; *miRs* microRNAs; *NC* negative control; *qPCR* quantitative polymerase chain reaction; *RNA* ribonucleic acid; *SIRT*1 silent information regulator 1; *SP* substance P; SRT1720, SIRT1-activator; *TGs* trigeminal ganglia; *URB*937, peripheral fatty-acid amide hydrolase; *WB* western blotting

## Conclusions and Future Perspectives

Over the past few years, the value of microRNAs in the diagnosis and therapy of multiple diseases has been extensively studied. However, the number of research regarding the role of miRNAs in migraine remains relatively small. Different clinical and preclinical studies have demonstrated the dysregulation of multiple miRNAs in migraineurs. As demonstrated, the most studied and, therefore, promising biomarker appears to be miR-34a, followed by miR-382 and miR-155, which indicates prospectively the main direction for more in-depth research. As all three have been scrutinized in clinical and preclinical studies, their putative value has grown in a wide area of migraine management. Moreover, that prompts to perform adequately detailed measurements within diverse study cohorts.

Furthermore, the alterations in the levels of investigated biomarkers were observed according to the type of migraine, the phase of the disease, and the applied treatment. Thus, the profiles of patients presenting the most significant changes in particular miRNA levels can be indicated. This may be essential for establishing novel diagnostic methods or even commencing studies focusing on the therapeutic use of miRNAs in specific groups of migraineurs.

**Fig. 3:**
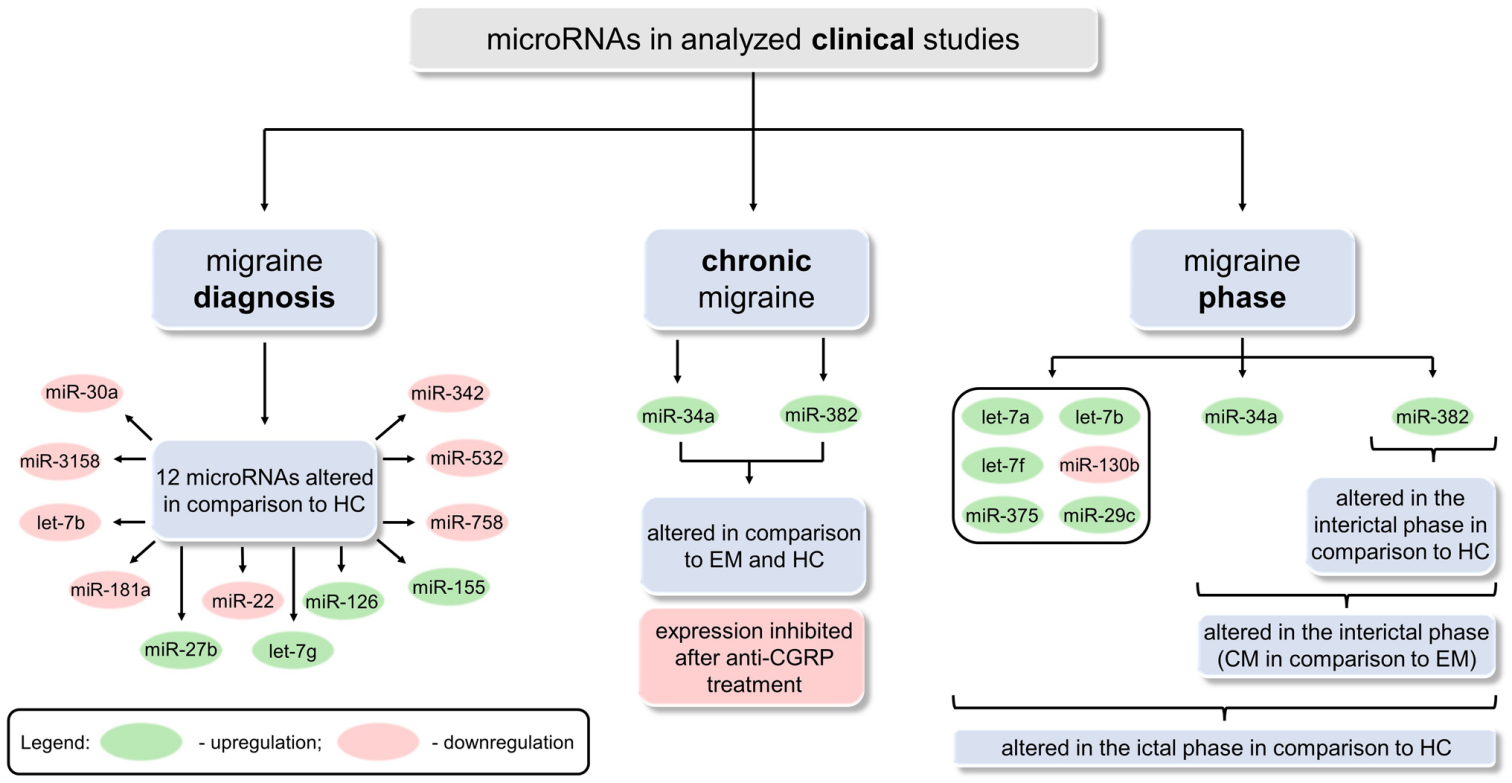
A graphical summary of the information included in clinical studies with the division into three parts: (i) migraine diagnosis, (ii) chronic migraine, and (iii) migraine phase. *CGRP* calcitonin gene-related peptide; *CM* chronic migraine; *EM* episodic migraine; *HC* healthy controls; *miR* microRNA; *RNA* ribonucleic acid

Performing a thorough analysis of all preclinical studies (Fig. 3) allows us to indicate multiple microRNAs as putative biomarkers in migraine in comparison to healthy controls. The differences in miRNA levels (upregulation/downregulation) result from different target genes encoding either inflammatory or anti-inflammatory proteins. Noteworthily, a trend (increase/decrease) for almost every miRNA remains the same in independent studies. Upregulated miRNAs may significantly influence neuroinflammation and underlying mechanisms of migraine pathogenesis by inhibiting their target genes, which are involved in suppressing inflammation. On the contrary, the downregulation of other miRNAs may increase the level of proteins responsible for inflammatory response. In turn, microRNAs are shown to be directly involved in the inflammatory process in migraine pathogenesis. The only miRNA presenting different expression in two independent studies was let-7b. However, according to Harmonizome 3.0 Database (Rouillard et al. 2016), genes encoding both inflammatory and anti-inflammatory proteins can be found among over 130 predicted target genes of mentioned miRNA. This may be a reasonable explanation for the observed inconsistency. Noteworthily, this observation shows that miRNAs which target genes encode proteins with different impacts on the inflammatory response, may have limited use as putative biomarkers.

Nowadays, an intensively developing field of migraine management is chronic migraine, and novel therapies are continually emerging. Therefore, we have found that as one of the main objects of interest in this systemic review and scrupulously analyzed all available data. Two miRNAs were shown as promising biomarkers in differentiating between chronic and episodic migraine, which still poses difficulties in everyday practice. Clinical manifestation may be inconclusive and difficult to assess clearly; therefore, a biochemical diagnostic marker is highly needed. The chronic migraine diagnosis is based on at least 3 months observation, indicating a valuable biomarker would be an undeniable achievement in this field, significantly shortening the diagnostic process. Thus, this is undoubtedly an area to focus on in further research.

**Fig. 4:**
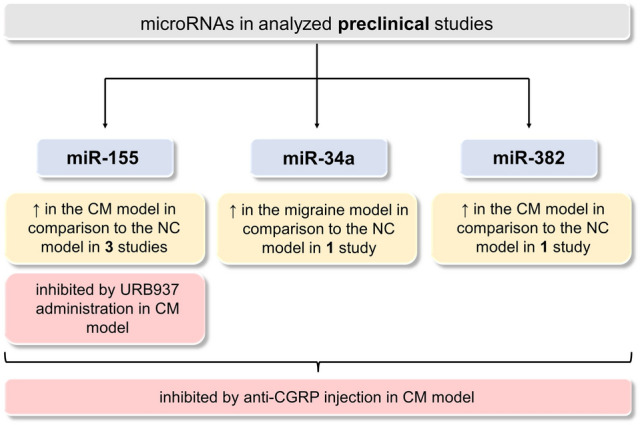
A graphical summary of the information included in preclinical studies showing the dysregulation patterns of three microRNAs (miR-155, miR-34a, miR-382). ↑, increase; *CGRP* calcitonin gene-related peptide; *CM* chronic migraine; *miR* microRNA; *NC* negative control; *RNA* ribonucleic acid; *URB*937, peripheral fatty-acid amide hydrolase

Furthermore, preclinical studies (Fig. 4) show not only the possibility of miRNAs’ use as diagnostic biomarkers but also their potential value in migraine therapy. In experimental models, levels of three miRNAs (miR-155, miR-34a, miR-382) were measured with additional assessments and interventions described in detail above. Preclinical studies confirmed a correlation between levels of several miRNAs and CGRP expression observed previously in clinical studies. Noteworthily, in recent years, the role of CGRP and 5-HT1F receptor (5-hydroxytryptamine receptor 1F) have been extensively investigated, resulting in several breakthroughs in migraine treatment, including chronic migraine.

Interestingly, interventions performed in preclinical studies with miRNA agonists or antagonists have been shown to influence CGRP levels and inflammation. Hopefully, in further studies, miRNAs may arise as another promising therapeutic target.

To summarize all the above, this study shows the significance of miRNAs in migraine. Further research in this field may lead to novel developments and hence, the improvement of disease management.

## Limitations

Undoubtedly the results of all the analyzed studies are highly promising and encourage further investigations. However, the number of clinical and preclinical research, especially the latter, remains very limited. Secondly, the number of patients enrolled in the clinical trials is relatively small, and there is still a lack of large multicenter studies. Finally, there is an increasing need for research that will not only focus on the diagnostic value of microRNAs in migraine but also probe into their therapeutic potential.

In conclusion, the role of miRNA in migraine requires extensive research involving larger groups of patients and focusing more on therapeutic options. However, that provides hope of a breakthrough in broadly understood migraine management.

## Data Availability

All data analysed during this study are included in this published article.
